# Presentation of Patients With Congenital Anomalies of the Kidney and Urinary Tract and *PAX2* Loss-of-Function Variants and Implications for Clinical Management

**DOI:** 10.1016/j.ekir.2025.08.037

**Published:** 2025-09-03

**Authors:** Leonie Greipel, Helge Martens, Lina Werfel, Ann Christin Gjerstad, Bernd Auber, Robert Geffers, Jan H. Bräsen, Augustina Jankauskiene, Anna Bjerre, Nele Kanzelmeyer, Dieter Haffner, Ruthild G. Weber

**Affiliations:** 1 Department of Human Genetics, Hannover Medical School, Hannover, Germany; 2 Department of Pediatric Kidney, Liver, Metabolic and Neurological Diseases, Hannover Medical School, Hannover, Germany; 3 PRACTIS Clinician Scientist Program, Dean’s Office for Academic Career Development, Hannover Medical School, Hannover, Germany; 4 Division of Paediatric and Adolescent Medicine, Oslo University Hospital, Oslo, Norway; 5 Genome Analytics Research Group, Helmholtz Centre for Infection Research, Braunschweig, Germany; 6 Nephropathology, Institute of Pathology, Hannover Medical School, Hannover, Germany; 7 Pediatric Center, Institute of Clinical Medicine, Vilnius University, Vilnius, Lithuania

**Keywords:** albuminuria, antiproteinuric measures, dominant-negative effect, kidney hypo-/dysplasia, living related donor evaluation, *PAX2*

## Abstract

**Introduction:**

*PAX2* variants, particularly loss-of-function (LOF) variants, can cause congenital anomalies of the kidney and urinary tract (CAKUT), mostly associated with renal coloboma syndrome (RCS), and focal segmental glomerulosclerosis (FSGS) marked by proteinuria.

**Methods:**

Whole-exome sequencing (WES) was performed in 301 pediatric patients with CAKUT. Deep phenotyping was done in 7 carriers of a *PAX2* LOF variant. The kidney phenotype was compared in pediatric patients with CAKUT and *PAX2* LOF variants (*n* = 104), compiled from our cohort (*n* = 7) and 12 publications (*n* = 97), and in those with wildtype *PAX2* from our cohort (*n* = 294). Genotype-phenotype correlations were explored.

**Results:**

Heterozygous inherited or *de novo PAX2* LOF variants were detected in 7 of 301 patients (2.3%), all presenting with bilateral (cystic) kidney hypoplasia/dysplasia/hypodysplasia (KHD). Full penetrance for a kidney phenotype, but variable expressivity was observed in our 10 carriers of a *PAX2* LOF variant, including parents who were not necessarily affected by CAKUT but by albuminuria or FSGS. In 104 pediatric carriers of a *PAX2* LOF variant with CAKUT, hallmark kidney manifestations were (cystic) KHD (97% vs. 59% in patients with CAKUT and wildtype *PAX2*, *P* < 0.0001) and albuminuria (significantly more severe than in patients with (cystic) KHD and wildtype *PAX2*, *P* < 0.0001), suggesting a proteinuric effect of *PAX2* LOF variants. Severe kidney anomalies, that is, cystic KHD or agenesis, were significantly more frequent in patients carrying the NM_000278.5(*PAX2*):c.76dupG variant in exon 2 with a possible dominant-negative effect than in patients with nonsense or frameshift variants in exon 3 to 7.

**Conclusion:**

In patients with CAKUT and *PAX2* LOF variants, close monitoring and antiproteinuric measures should be considered, and *PAX2* variant testing is recommended in living related donors.

## Graphical abstract

**Figure:**
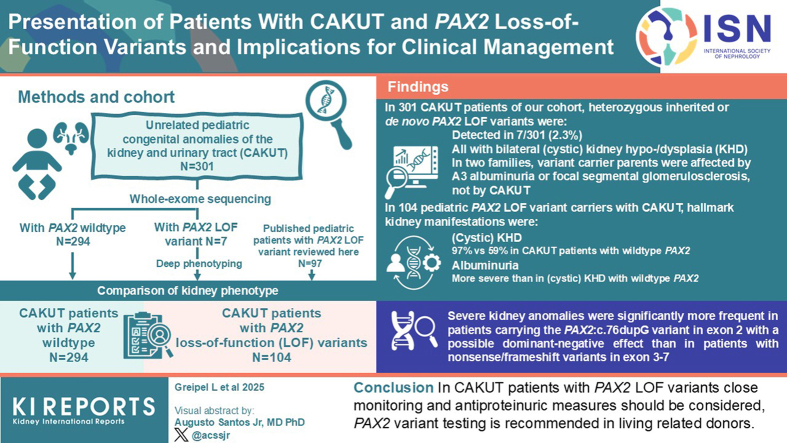

See Commentary on Page 3744

*PAX2* encodes paired box 2, a transcription factor involved in GDNF/RET signaling with a pivotal role in kidney development,^1^ including the regulation of ureteric bud outgrowth, branching, and nephron differentiation.^2^, ^3^, ^4^ In addition to the kidney, *PAX2* is expressed in the eye, ear, and central nervous system during development.^5^^,^^6^ Consequently, heterozygous *PAX2* variants can cause kidney and eye anomalies as well as hearing loss, an autosomal dominant disorder known as RCS or papillorenal syndrome.^5^, ^6^, ^7^ Mice with a heterozygous *PAX2* LOF variant present with developmental defects, including kidney manifestations, similar to human RCS^8^ with increased apoptosis and reduced branching of the ureteric bud in fetal kidneys.^9^ Abnormal kidney structure or function have been reported in 92% of patients with RCS, with kidney hypodysplasia as the most common finding.^6^ *PAX2* variation has also been associated with isolated CAKUT and, more recently, with FSGS.^10^, ^11^, ^12^ In patients with CAKUT, *PAX2* is one of the most frequently mutated genes.^13^^,^^14^ LOF variants, such as the common NM_000278.5(*PAX2*):c.76dupG, represent the most prevalent pathogenic *PAX2* variant type reported in RCS,^6^^,^^15^ underlining the importance of *PAX2* LOF variants in CAKUT.

Therefore, this study exclusively focused on pediatric patients with CAKUT and *PAX2* LOF variants with the aim of gaining insights into their kidney phenotype and function, to explore genotype-phenotype correlations and clinical implications. We identified and characterized carriers of a *PAX2* LOF variant in our own cohort of CAKUT families, compiled a large number of pediatric carriers of a *PAX2* LOF variant with CAKUT through literature review, and compared certain parameters with our patients with CAKUT carrying wildtype *PAX2*.

## Methods

### Patient Characterization

This study was approved by the Ethics Boards of Hannover Medical School, Hannover, Germany, Oslo University Hospital, Oslo, Norway, and Vilnius University, Vilnius, Lithuania. Each family gave their informed consent for participating in this study. All pediatric patients with CAKUT (except for patients with isolated vesicoureteral reflux) presenting to the participating pediatricians in Hannover from May 2012 to October 2023, in Oslo from October 2013 to September 2022, and in Vilnius from June 2023 to October 2023 who consented were included in the study. A total of 301 unrelated pediatric patients with CAKUT aged 0 to 18 years were analyzed, of which 105 were female (35%) and 196 were male (65%). CAKUT phenotypes included KHD not combined with posterior urethral valves (PUV), cystic KHD (comprising unilateral or bilateral cystic dysplasia/hypodysplasia, multicystic dysplastic kidney, and solitary kidney cysts) not combined with PUV, kidney agenesis (KA), duplex kidney, horseshoe kidney, hydronephrosis, ureter anomalies, as well as PUV with and without ureter or kidney anomalies.

Stages of chronic kidney disease (CKD) were determined according to the Kidney Disease: Improving Global Outcomes 2024 guidelines.^16^ Z-scores for total native kidney volume determined using ultrasound were calculated using age-related and gender-related reference values,^17^ and the estimated glomerular filtration rate was calculated using the revised Schwartz formula.^18^ The urine albumin-to-creatinine ratio (uACR) in g/mol was calculated to determine albuminuria. Sections from a nephrectomy specimen of patient B005-II.02 were stained with hematoxylin-eosin, and immunohistochemistry was done using a PAX2 rabbit monoclonal antibody (EP235; Cell Marque Corporation, Rocklin, CA).

### Literature Review

We conducted a comprehensive literature search to obtain genotype and phenotype data of published pediatric patients with CAKUT and *PAX2* LOF variants. The literature search was conducted in the PubMed database using the terms “PAX2 and congenital anomalies of the kidney and urinary tract or renal coloboma syndrome,” “PAX2 and children,” and “CKD and pediatrics and congenital anomalies of the kidney and urinary tract,” and limited to reports in English published between December 2011 and February 2025. Publications with insufficient information on the CAKUT phenotype or kidney function, and reports of patients with isolated vesicoureteral reflux or age >18 years were excluded. Reported values for proteinuria or albuminuria in carriers of a *PAX2* variant were converted into uACR in g/mol according to Rees *et al.*^19^

### DNA Extraction, WES, and Targeted Sequencing

DNA was extracted from peripheral blood or urine of CAKUT families of our cohort using QIAmp DNA Blood Maxi Kit (Qiagen, Hilden, Germany) or Quick-DNA Urine Kit (ZYMO Research Europe GmbH, Freiburg, Germany). For WES of 301 unrelated pediatric patients with CAKUT of our cohort, SureSelectXT Human All Exon (Agilent, Santa Clara, CA) or IDT xGen Exome Research Panel v2 (Integrated DNA Technologies, Coralville, IA) enrichment kits and a HiSeq or NovaSeq sequencer (Illumina, San Diego, CA) were used. The mean target coverage was at least 50×. The human reference genome build used for alignment was hg38/GRCh38, variations were called using CLC Genomic Workbench (version 23.0.1, Qiagen) and annotated and prioritized using Clinical Insight Interpret Translational (Qiagen). Quality filters were applied (coverage ≥15, call quality ≥ 50, allele fraction ≥ 30), and *PAX2* (NM_000278.5) LOF variants, that is, frameshift, out-of-frame insertions/deletions, stop gained/lost, and splice site variants up to 5 bp into the intron, were retained. The minor allele frequencies of variants were retrieved from the Genome Aggregation Database (gnomAD v4.1.0). ClinVar,^20^ HGMD,^21^ SpliceAI,^22^ and the American College of Medical Genetics and Genomics / Association for Molecular Pathology guidelines and updates^23^^,^^24^ were used for variant assessment. Oligonucleotides used for amplification of genomic DNA and targeted sequencing to verify variants detected by WES and determine familial segregation are presented in Supplementary Table S1 and the Supplementary References.

### RNA Extraction, Reverse Transcription, and cDNA Sequencing

Total RNA was extracted from urine samples of patients B061-I.02, N075-III.03, and C018-II.01, using the Quick-RNA Miniprep Plus Kit (ZYMO Research Europe GmbH). Digestion of possibly present genomic DNA was performed using DNase I (50 U/μl; ZYMO Research Europe GmbH). Subsequently, cDNA was generated using the First-Strand cDNA synthesis protocol of the SuperScript III or IV Reverse Transcriptase Kit (Invitrogen, Thermo Fisher Scientific, Waltham, MA). cDNA was amplified using Platinum Taq DNA polymerase (Thermo Fisher Scientific) or Taq DNA Polymerase kit (Qiagen) and exon spanning oligonucleotides (Supplementary Table S1) to exclude amplification of possibly remaining genomic DNA.

### Statistical Analysis

All statistics were done using MATLAB 2022b (The MathWorks, Inc., Natick, MA) and GraphPad Prism, version 10.2.0 (GraphPad Software, San Diego, CA). Normal distribution of data was evaluated using the Kolmogorov-Smirnov test. Between group differences were analyzed using the Fisher Exact test. Within group differences were analyzed using the unpaired *t* test. The Mann-Whitney U test was used for nonnormally distributed values. *P*-values < 0.05 were considered significant.

## Results

### Heterozygous *PAX2* LOF Variants Were Identified in 7 of 301 Pediatric Patients With CAKUT (2.3%) in our Cohort

**Figure 1:**
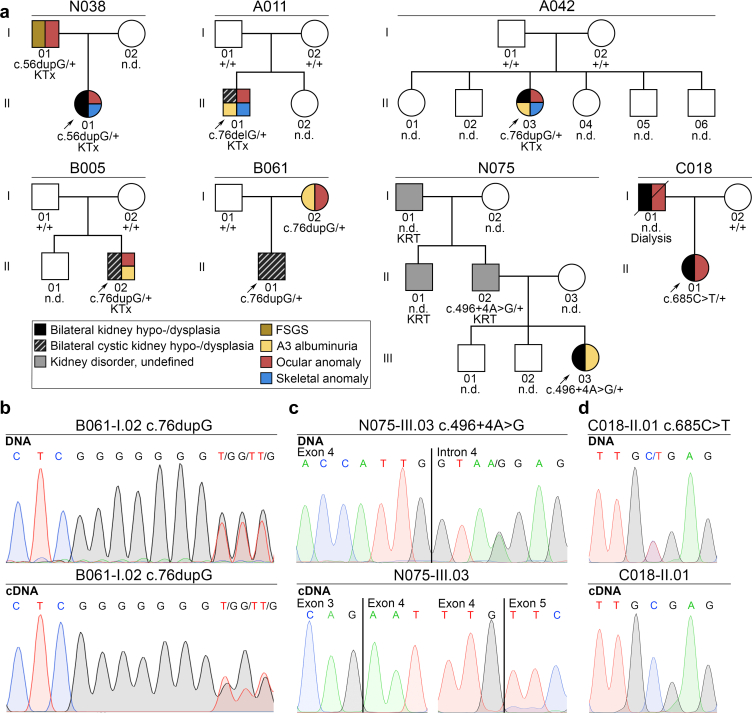
Pedigrees of our pediatric patients with CAKUT carrying a *PAX2* LOF variant, and electropherograms of DNA and cDNA analysis of patients with available urine RNA. (a) In our cohort, 7 of 301 unrelated pediatric index patients (indicated by a black arrow), who were all affected by bilateral (cystic) KHD, carried a heterozygous *PAX2* LOF variant. Variant carrying parents could be affected by FSGS or A3 albuminuria, not CAKUT. Carrier status is also assumed in the deceased father of patient C018-II.01 who had presented with bilateral KHD and myopia. Three *PAX2* LOF variants were *de novo*. Squares represent males, circles females, open symbols unaffected individuals, and filled symbols individuals affected by anomalies as indicated in the figure. (b) The NM_000278.5(*PAX2*):c.76dupG variant was detected on leukocyte DNA (top) and on cDNA transcribed from urine mRNA (bottom) of patient B061-I.02. This finding indicates that the mRNA with this variant is not degraded by NMD but is predicted to result in a severely truncated nonfunctional PAX2 protein with a possible dominant-negative effect. (c and d) Both (c) the splice region variant NM_000278.5(*PAX2*):c.496+4A>G predicted to affect both splice sites of exon 4, and (d) the nonsense variant NM_000278.5(*PAX2*):c.685C>T were found on leukocyte DNA (top), but no change was found on cDNA transcribed from urine mRNA (bottom) of patients (c) N075-III.03 or (d) C018-II.01, respectively, indicating NMD of the altered mRNAs resulting in haploinsufficiency. +, *PAX2* wildtype sequence; CAKUT, congenital anomalies of the kidney and urinary tract; FSGS, focal segmental glomerulosclerosis; KHD, kidney hypoplasia/dysplasia/hypodysplasia; KRT, kidney replacement therapy; KTx, kidney transplantation; LOF, loss-of-function; n.d., DNA was not available; NMD, nonsense-mediated RNA decay.

In 7 of 301 unrelated patients with CAKUT (2.3%), WES yielded 5 different *PAX2* LOF variants, that is, 3 different frameshift variants, 1 splice-site, and 1 nonsense variant (Table 1^25^, ^26^, ^27^, ^28^, ^29^, Figure 1, and Supplementary Figure S1). The NM_000278.5(*PAX2*):c.76dupG variant was identified in 3 patients, whereas the c.56dupG, c.76delG, c.496+4A>G, and c.685C>T variants were found in 1 patient each (case reports of all variant carriers are provided in the Supplementary Results). All variants were rare (c.56dupG and c.496+4A>G were novel), heterozygous and classified as likely pathogenic or pathogenic using the American College of Medical Genetics and Genomics / Association for Molecular Pathology guidelines (Table 1). For the c.496+4A>G variant, SpliceAI predicted a decreased probability that the 3’ and 5’ splice sites of exon 4 are used as splice donor and acceptor. In 3 of 7 patients (43%), the *PAX2* variants occurred *de novo*. The *PAX2* variants were inherited in 3 of 7 patients (43%) and presumably also in case C018-II.01, in which the father could not be genetically tested because he had passed away at the age of 40 years on dialysis, having presented with an almost identical kidney and ocular phenotype as his daughter carrying the *PAX2* nonsense variant (Table 1 and Figure 1a).

**Table 1:** Rare heterozygous *PAX2* (NM_000278.5) LOF variants identified in 7 of 301 pediatric patients with CAKUT (2.3%) of our cohort

Case, gender, country of origin	Nucleotide change	Deduced protein change	Inheritance	MAF (study cohort)	MAF (control cohort^a^)	Variant frequency comparison of study vs. control cohort	ClinVar^b^ (phenotype)	HGMD^c^	SpliceAI^d^ score	ACMG/ AMP classification^e^	Detected on cDNA (consequence)	References
N038-II.01, female, Ghana/UK	c.56dupG	p.(Val20fs∗34)	Pat	0.0017	0.00	*P* = 0.0004	-	-	0.08	P	ND (prediction: no NMD, truncated protein)	-
A011-II.01, male, Germany	c.76delG	p.(Val26fs∗3)	DNV^f^	0.0017	0.000001241	*P* = 0.0011	P (RCS/ FSGS)	DM	0.03	P	ND (prediction: no NMD, truncated protein)	Bower *et al.*^6^, Schimmenti *et al.*^7^, Kosfeld *et al.*^25^, Werfel *et al.*^26^
A042-II.03, female, Lybia	c.76dupG	p.(Val26fs∗28)	DNV	0.0050	0.000004965	*P* < 0.0001	P (RCS/ FSGS)	DM	0.14	P	Yes (no NMD, truncated protein predicted)	Bower *et al.*^6^, Schimmenti *et al.*^7^, Werfel *et al.*^26^
B005-II.02, male, Germany	c.76dupG	p.(Val26fs∗28)	DNV	0.0050	0.000004965	*P* < 0.0001	P (RCS/FSGS)	DM	0.14	P	Yes (no NMD, truncated protein predicted)	Bower *et al.*^6^, Schimmenti *et al.*^7^, Werfel *et al.*^26^
B061-II.01, male, Germany	c.76dupG	p.(Val26fs∗28)	Mat	0.0050	0.000004965	*P* < 0.0001	P (RCS/FSGS)	DM	0.14	P	Yes (no NMD, truncated protein predicted)	Bower *et al.*^6^, Schimmenti *et al.*^7^, Werfel *et al.*^26^
N075-III.03, female, Pakistan	c.496+4A>G	-	Pat	0.0017	0.00	*P* = 0.0004	-	-	0.77 (dl), 0.69 (al)	LP	No (NMD)	-
C018-II.01, female, Germany	c.685C>T	p.(Arg229∗)	Not mat, probably pat	0.0017	0.00	*P* = 0.0004	P (RCS/ FSGS)	DM	0.00	P	No (NMD)	Yang *et al.*^27^, Xiong *et al.*^28^, Liu *et al.*^29^

ACMG/AMP, American College of Medical Genetics and Genomics/Association for Molecular Pathology; al, acceptor loss predicted; CAKUT, congenital anomalies of the kidney and urinary tract; dl, donor loss predicted; DM, disease-causing variant; DNV, *de novo* variant; FSGS, focal segmental glomerulosclerosis; gnomAD, genome aggregation database; LP, likely pathogenic; MAF, minor allele frequency; mat, maternal; ND, not determined; NMD, nonsense mediated decay; P, pathogenic; pat, paternal; RCS, renal coloboma syndrome.

a gnomAD v4.1.0 total population (https://gnomad.broadinstitute.org).

b ClinVar,^20^ database of the NIH National Library of Medicine that aggregates information about genomic variation and its relationship to human health (https://www.ncbi.nlm.nih.gov/clinvar).

c HGMD,^21^ the Human Gene Mutation Database (https://www.hgmd.cf.ac.uk/ac/index.php).

d SpliceAI^22^ (https://spliceailookup.broadinstitute.org).

e According to Richards et al.^23^ and Abou Tayoun et al.^24^

f Confirmed paternity/maternity.

### Of the *PAX2* LOF Variants Detected in our Cohort, the c.76dupG Variant Escapes, but the c.496+4A>G and c.685C>T Variants are Subjected to Nonsense-Mediated RNA Decay

To determine whether the LOF variants detected in our cohort escape nonsense-mediated RNA decay (NMD) and result in a truncated PAX2 protein or are degraded by NMD, cDNA generated from urine mRNA of carriers of a *PAX2* LOF variant was sequenced, if available. The c.76dupG variant was detected on cDNA (Figure 1b), indicating that it escapes NMD, as might be expected because the premature termination codon is predicted to be introduced within 162 nucleotides of the translation initiation codon, and at such a location typically does not trigger NMD.^30^ The same prediction holds true for the c.56dupG variant resulting in a premature termination codon at the same position. Thus, the c.56dupG and c.76dupG are predicted to result in a truncated protein of 54 amino acids containing a part of the DNA-binding paired domain, but not the octapeptide, homeodomain, or transactivation domain, which could have a dominant-negative effect. In contrast, no altered exon 4 boundary or c.685C>T variant was detected on cDNA of patients carrying the c.496+4A>G or c.685C>T variants, respectively, indicating that these variants elicit NMD and cause haploinsufficiency (Figure 1c and d).

### Bilateral (Cystic) KHD, Kidney Failure, and Severe Albuminuria Were Frequent Features of the 7 Pediatric Carriers of a *PAX2* LOF Variant in our Cohort

**Figure 2:**
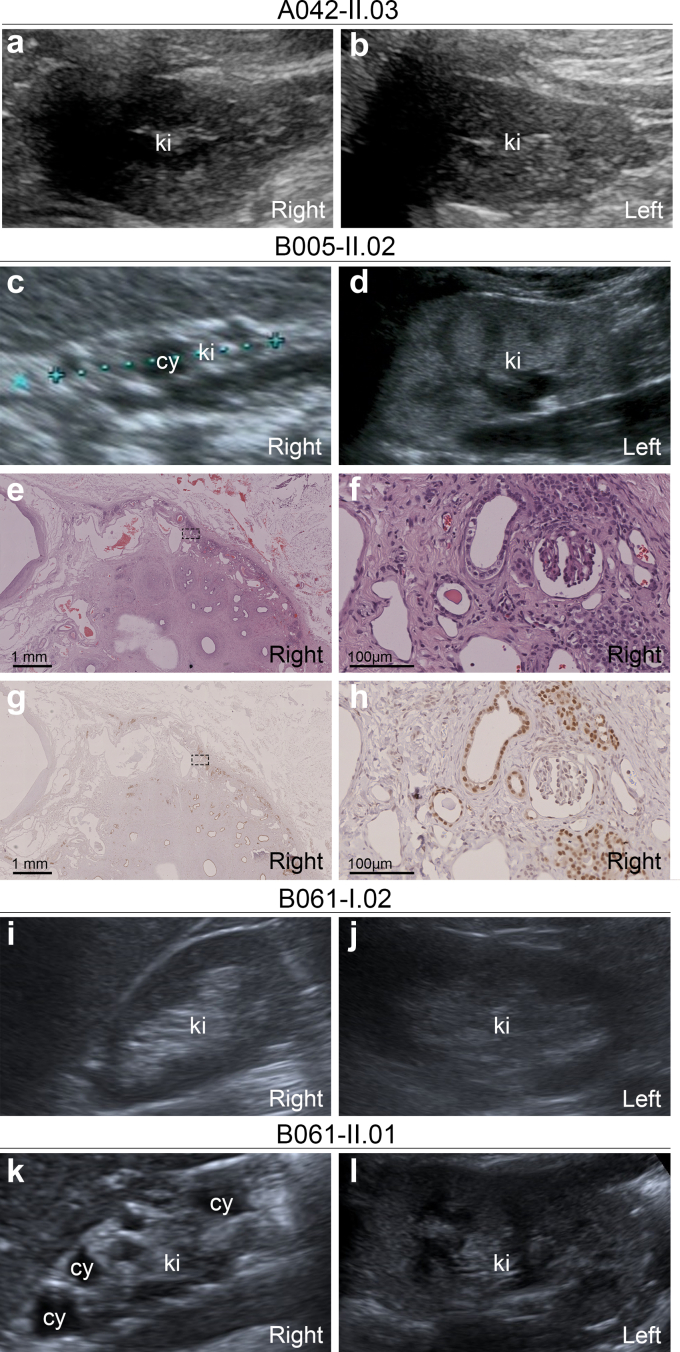
Kidney phenotypes of our patients carrying the NM_000278.5(*PAX2*):c.76dupG variant. (a and b) Patient A042-II.03 was diagnosed with bilateral KHD without cysts using ultrasound. (c–h) Patient B005-II.02 presented with (c) right-sided cystic KHD, and (d) left-sided KHD without cysts using ultrasound. (e and f) Hematoxylin-eosin and (g and h) PAX2 staining of kidney sections obtained after nephrectomy confirmed severe right-sided cystic KHD. (i and j) The kidneys of B061-I.02, the mother of index patient B061-II.01, who turned out to be a *PAX2* LOF variant carrier, were sonographically unremarkable. A3 albuminuria was detected by kidney work-up done after a *PAX2* LOF variant was identified in her son. (k and l) Patient B061-II.01 presented with (k) right-sided MCDK and (l) left-sided KHD diagnosed using ultrasound. cy, cyst; KHD, kidney hypoplasia/dysplasia/hypodysplasia; ki, kidney; LOF, loss-of-function; MCDK, multicystic dysplastic kidney.

By deep phenotyping, the 7 patients with CAKUT carrying a *PAX2* LOF variant presented with bilateral (cystic) KHD (Figure 1, Figure 2, and Table 2), accounting for 7 of 105 patients (6.7%) with the diagnosis of bilateral (cystic) KHD that was not combined with PUV. Unilateral cysts were present in 3 of 7 patients (43%) with KHD and a *PAX2* LOF variant (Figure 2 and Table 2). In one such case, a nephrectomy specimen of the right-sided hypodysplastic cystic kidney was available, and hematoxylin-eosin and PAX2 staining showed very little morphologically normal kidney tissue indicating severe dysplasia (Figure 2e–h). The most common extrarenal features of pediatric patients with CAKUT and a *PAX2* LOF variant were ocular anomalies in 5 of 7 cases (71%) (Figure 1 and Table 2).

**Table 2:** Clinical characteristics of pediatric patients with CAKUT and their relatives carrying a *PAX2* LOF variant of our cohort

Case	*PAX2*^a^ variant, inheritance	WG/birthweight (g)	Age at last follow-up (yr)	Kidney phenotype	Albuminuria^b^ before KTx	CKD stage^b^	UTI/prophylaxis	Age at KTx / waiting list for KTx	Post-KTx diagnosis	Extrarenal phenotype	Phenotype in other family member(s) with a (presumed) *PAX2* LOF variant
N038-II.01	c.56dupG p.(Val20fs∗34), pat	38 / 3346	18	KHD (r+l)	NA	G5t	No / no	4 yr 3 mo / NA	Chronic rejection, re-KTx (9 yr), overweight, hypertension	Severe myopia, macrocephaly, high-arched palate, short narrow palpebral fissures	Father: FSGS, adult-age KTx, visual impairment
A011-II.01	c.76delG p.(Val26fs∗3), DNV	33 / 1200	18	KHD (r+l), single cyst (r)	A3	G5t	No / no	4 yr 9 mo / 1 yr 11 mo	Ogilvie syndrome, PTLD, recurrent pneumonia, 2 rejections, pancreatitis	Premature infant, severe hyperopia, pes calcaneus (r+l)	-
A042-II.03	c.76dupG p.(Val26fs∗28), DNV	NA	18	KHD (r+l), hydronephrosis grade 1 (r)	A3	G5t	Yes / no	15 yr 11 mo / 14 yr 11 mo	Glucose intolerance, non-HLA / HLA class II antibodies	Severe myopia, luxation of the hip, abdominal hernia	-
B005-II.02	c.76dupG p.(Val26fs∗28), DNV	37 + 0 / 2990	15	KHD (r+l), with multiple cysts (r), VUR (r+l)	A3	G5t	Yes / yes, since age of 1 yr	9 yr 4 mo / 8 yr 6 mo	Hepatitis E	Optic nerve dysplasia (r+l), amaurosis (r), cryptorchidism	-
B061-II.01	c.76dupG p.(Val26fs∗28), mat	39 + 5 / 2720	4	KD (l), MCDK (r), VUR (r+l)	A2	G2	No / yes, since birth	-	-	None	Mother: A3 albuminuria, myopia
N075-III.03	c.496+4A>G, pat	36 + 4 / 3390	8	KHD (r+l), kidney biopsy: oligomeganephronia	A3	G3	No / no	-	-	None	Father: Kidney failure (16 yr); paternal uncle and grandfather: adult-age KRT for unknown reasons
C018-II.01	c.685C>T p.(Arg229∗), not mat, probably pat	41 + 0 / 3410	16	KD (r+l)	A2	G2	No / no	-	-	Myopia, G6PD, overweight	Father: KHD (r+l), dialysis (36 yr), myopia, G6PD

CAKUT, congenital anomalies of the kidney and urinary tract; CKD, chronic kidney disease; DNV, *de novo* variant; FSGS, focal segmental glomerulosclerosis; G6PD, glucose-6-phosphate dehydrogenase deficiency; HLA, human leukocyte antigen; KD, kidney dysplasia; KDIGO, Kidney Disease: Improving Global Outcomes; KHD, kidney hypodysplasia; KRT, kidney replacement therapy; KTx, kidney transplantation; l, left; mat, maternal; LOF, loss-of-function; MCDK, multicystic dysplastic kidney; mo, months; NA, not available; pat, paternal; PTLD, posttransplant lymphoproliferative disorder; r, right; t, transplanted; UTI, urinary tract infection; VUR, vesicoureteral reflux; WG, weeks of gestation.

a Reference sequence: NM_000278.5, genome build hg38/GRCh38.

b According to the KDIGO 2024 guidelines.^16^

**Figure 3:**
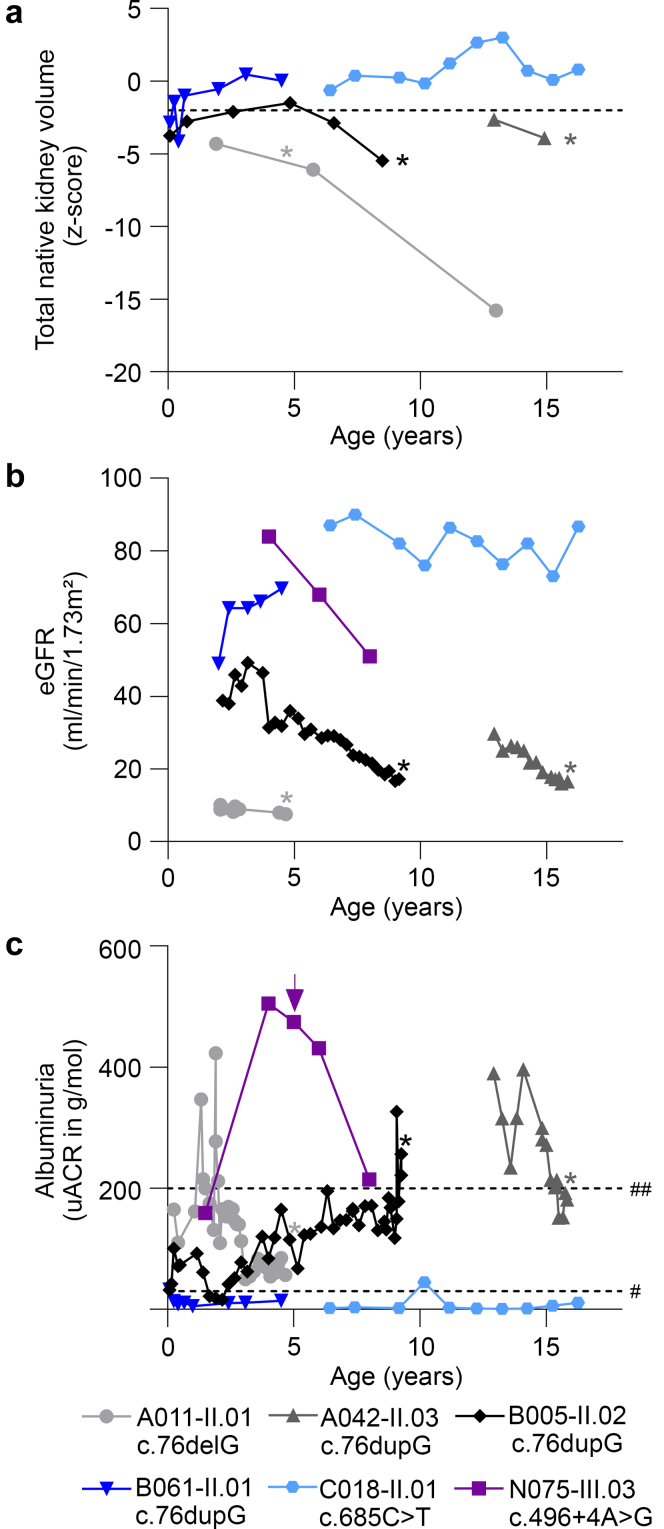
Kidney volume, eGFR and albuminuria as a function of age in our pediatric patients with CAKUT and a *PAX2* LOF variant. (a) Total native kidney volume was determined in 5 of 7 patients. Z-scores were calculated according to Obrycki *et al.*^17^ using an age-dependent formula and are shown as a function of age (time point of KTx indicated by an asterisk). Kidney hypoplasia is diagnosed with a z-score ≤ −2 (dotted line). (b and c) Kidney function was measured in 6 of 7 patients. (b) eGFR and (c) albuminuria are shown as a function of age until the patients received KTx (time point of KTx indicated by an asterisk). The level of albuminuria decreased in patient N075-III.03 after the start of angiotensin-converting enzyme inhibition (indicated by an arrow in c). A3 albuminuria (uACR > 30 g/mol), dashed line marked with #; albuminuria in the nephrotic range (uACR > 200 g/mol), dashed line marked with ##. CAKUT, congenital anomalies of the kidney and urinary tract; eGFR, estimated glomerular filtration rate; KTx, kidney transplantation; LOF, loss-of-function; uACR, urine albumin-to-creatinine ratio.

Kidney volume was low (z-score < −2) before kidney transplantation in 3 of 5 patients (60%) with *PAX2* LOF variants and available data, whereas 2 patients presented with normal kidney volume until their current age (4 and 16 years, Figure 3a). Kidney failure (CKD stage G5, i.e., estimated glomerular filtration rate < 15 ml/min per 1.73 m^2^) before the age of 16 years occurred in 4 of 5 patients (80%) with *PAX2* LOF variants who are currently ≥ 15 years (Table 2 and Figure 3b). Severe albuminuria (A3, i.e., > 30 g/mol) was observed in 4 of 6 cases (66%) with *PAX2* LOF variants and available data, that is, in 3 kidney transplantation patients and in 1 patient with CKD stage G3 at the age of 8 years (Table 2 and Figure 3c). The 2 patients with normal kidney volume had CKD stage G2 and A2 albuminuria at the age of 4 and 16 years (Table 2 and Figure 3).

Patient N075.III-03 had a decline in kidney function between the age of 4 years (estimated glomerular filtration rate of 84 ml/min per 1.73 m^2^) and 8 years (51 ml/min per 1.73 m^2^) (Figure 3b). Treatment with the angiotensin-converting enzyme inhibitor, enalapril was started at the age of 5 years (arrow, Figure 3c) resulting in decreased albuminuria (505 g/mol at the age of 4 years to 214 g/mol at the age of 8 years). The other patients did not receive any inhibitors of the renin-angiotensin-aldosterone system.

### Full Penetrance for a Kidney Phenotype but Variable Expressivity was Observed in Carriers of a *PAX2* LOF Variant in our Cohort

In our cohort, all carriers of a *PAX2* variant (7 pediatric patients and 3 parents) presented with a kidney phenotype, resulting in a penetrance of 100% (Figure 1). However, variable expressivity was also observed. Although 2 pediatric patients carried the same *PAX2* variant (c.76dupG) and presented with the same CAKUT phenotype (bilateral cystic KHD), their clinical course was quite different: at the age of 4 years, CKD stage G3b and A3 albuminuria of 84 g/mol (B005-II.02) or CKD stage G2 and A2 albuminuria of 14 g/mol (B061-II.01) (Table 2, and Figures 1 and 3). Two parents carrying *PAX2* LOF variants were not affected by CAKUT, although their child with the same variant was, but presented with either A3 albuminuria (44 g/mol, B061-I.02) or adult-onset kidney failure due to FSGS (N038-I.01) (Figure 1). In the former, A3 albuminuria was only diagnosed in a kidney work-up that was done following detection of the *PAX2* LOF variant first in her child, then in herself.

### KHD Without or With Cysts and Albuminuria Were Hallmarks of 104 Pediatric Patients With CAKUT Carrying a *PAX2* LOF Variant Compiled Here, Leading to Childhood-Onset Kidney Failure in Approximately Half of the Patients

**Figure 4:**
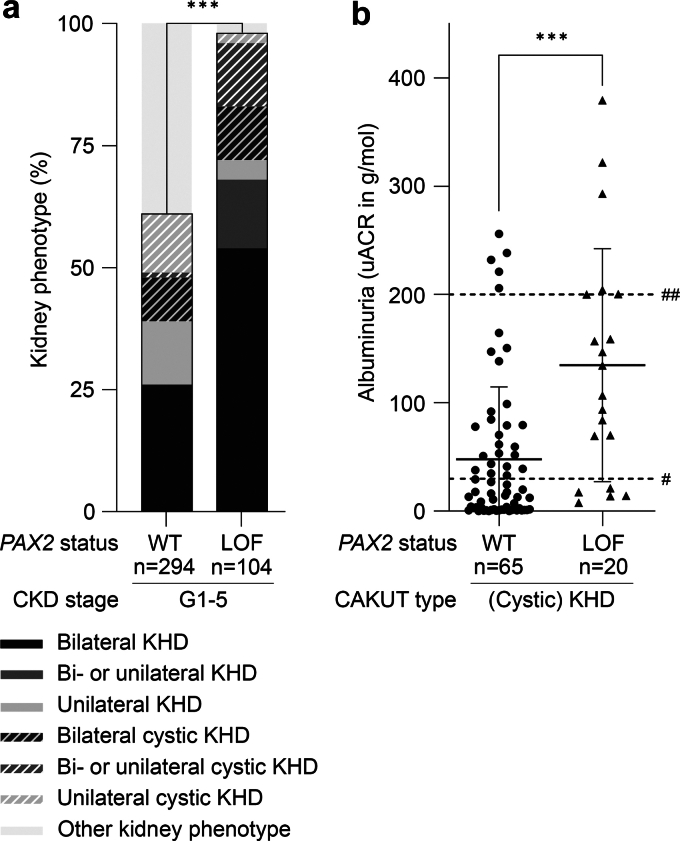
A comparison of the kidney phenotype and albuminuria levels in pediatric patients with CAKUT and without versus with a *PAX2* LOF variant yields 2 hallmark presentations of CAKUT due to *PAX2* LOF variants: (cystic) KHD and severe albuminuria. (a) The CAKUT phenotype almost exclusively observed in patients with *PAX2* LOF variants was bilateral or unilateral KHD without or with cysts including MCDK, referred to as (cystic) KHD. (Cystic) KHD was significantly more frequent in patients with all CKD stages (G1–G5) carrying a *PAX2* LOF variant compared with those with wildtype *PAX2*. (b) The level of albuminuria was significantly higher in patients with unilateral or bilateral (cystic) KHD carrying a *PAX2* LOF variant compared with those with wildtype *PAX2* (shown are uACRs with mean and SD). A3 albuminuria (uACR > 30 g/mol), dashed line marked with #; albuminuria in the nephrotic range (uACR > 200 g/mol), dashed line marked with ##; CAKUT, congenital anomalies of the kidney and urinary tract; CKD, chronic kidney disease; cystic KHD, all cystic KHD phenotypes including MCDK and solitary kidney cysts not combined with PUV; KHD, kidney hypoplasia/dysplasia/hypodysplasia not combined with PUV; LOF, loss-of-function; MCDK, multicystic dysplastic kidney; other kidney phenotype, all other kidney phenotypes including PUV with KHD; PUV, posterior urethral valves; SD, standard deviation; uACR, urine albumin-to-creatinine ratio; WT, wildtype. ∗∗∗*P* < 0.001.

We compiled the phenotypes of a total of 104 pediatric carriers of a *PAX2* LOF variant with CAKUT by additionally reviewing 12 previous publications (Supplementary Tables S2 and S3, Supplementary Figure S2).^15^^,^^27^, ^28^, ^29^^,^^31^, ^32^, ^33^, ^34^, ^35^, ^36^, ^37^, ^38^ Of these, 56 carriers of a *PAX2* LOF variant (54%) had kidney failure before the age of 18 years (Supplementary Table S2) with a median age of 9.5 years (range: 2 weeks–17.5 years). To identify CAKUT phenotypes typically associated with *PAX2* LOF variants, we compared these phenotype data with those of 294 CAKUT cases carrying wildtype *PAX2* from our cohort (Table 3, Supplementary Table S4, and Supplementary Figure S2). We found unilateral or bilateral (cystic) KHD not combined with PUV to be the hallmark CAKUT phenotype in carriers of a *PAX2* LOF variant with all CKD stages, because this CAKUT type was almost exclusively found and significantly more frequent in patients with versus those without *PAX2* LOF variants (101/104 [97%] vs. 174/294 [59%], *P* < 0.0001, Figure 4a and Table 3).

**Table 3:** Comparison of the clinical characteristics of pediatric patients with CAKUT and *PAX2* wildtype versus a *PAX2* LOF variant

Characteristic		Pediatric patients with CAKUT and		Comparison (*P*-value)
		*PAX2* wildtype^a^	*PAX2* LOF variant^b^	
Age (yrs) at last follow-up	Median (range)	13.5 (0–18) (*n* = 294)	9.6 (0.003–18) (*n* = 102)	< 0.0001
Gender	Female	101/294 (34.4%)	40/104 (38%)	0.4753
	Male	193/294 (65.6%)	64/104 (62%)	0.4753
CAKUT type standardized	All KHD	110/294 (37.4%)	75/104 (72.1%)	< 0.0001
	• Bilateral KHD	• 72/294 (24.5%)	• 56/104 (53.85%)	< 0.0001
	• Bi-/unilateral KHD	• 0/294 (0%)	• 15/104 (14.4%)	< 0.0001
	• Unilateral KHD	• 38/294 (12.9%)	• 4/104 (3.85%)	0.0087
	All cystic KHD	64/294 (21.8%)	26/104 (25%)	0.4980
	• Bilateral cystic KHD	• 26/294 (8.8%)	• 11/104 (10.6%)	0.5627
	• Bi-/unilateral cystic KHD	• 2/294 (0.7%)	• 13/104 (12.5%)	< 0.0001
	• Unilateral cystic KHD	• 36/294 (12.3%)	• 2/104 (1.9%)	0.0014
	Other	120/294 (40.8%)	3/104 (2.9%)	< 0.0001
CKD stage^c^ at last follow-up	G1	73/294 (24.8%)	1/104 (1.0%)	< 0.0001
	G2	44/294 (15.0%)	8/104 (7.7%)	0.0634
	G3a	11/294 (3.8%)	7/104 (6.7%)	0.2694
	G3b	17/294 (5.8%)	2/104 (1.9%)	0.1780
	G3a/b	3/294 (1.0%)	7/104 (6.7%)	0.0041
	G4	13/294 (4.4%)	14/104 (13.5%)	0.0030
	G5	133/294 (45.2%)	56/104 (53.8%)	0.1388
	ND	0/294 (0.0%)	9/104 (8.7%)	< 0.0001
Age (yrs) at CKD stage G5/KRT	Median (range)	4.0 (0.0–17.9) (*n* = 133)	9.5 (0.04–17.5) (*n* = 37)	0.0003
Albuminuria^c^ before KTx	A1	29/104 (27.9%)	0/20 (0.0%)	0.0036
	A2	25/104 (24.0%)	5/20 (25.0%)	1.0000
	A3	50/104 (48.1%)	15/20 (75.0%)	0.0303
uACR (g/mol) before KTx	Median (range)	25.7 (0.0–291.4) (*n* = 104)	120.7 (7.7–379.4) (*n* = 20)	< 0.0001

CAKUT, congenital anomalies of the kidney and urinary tract; cystic KHD, all cystic KHD phenotypes including multicystic dysplastic kidney and solitary kidney cysts not combined with posterior urethral valves; KDIGO, Kidney Disease: Improving Global Outcomes; KHD, kidney hypoplasia/dysplasia/hypodysplasia not combined with posterior urethral valves; KRT, kidney replacement therapy; KTx, kidney transplantation; LOF, loss-of-function; ND, not defined; uACR, urine albumin-to-creatinine ratio.

a Data from our CAKUT cohort.

b Data from our CAKUT cohort and 12 publications.^15^^,^^27^, ^28^, ^29^^,^^31^, ^32^, ^33^, ^34^, ^35^, ^36^, ^37^, ^38^

c According to the KDIGO 2024 guidelines.^16^

We noted that proteinuria was present in 38 of 47 patients (81%) with CAKUT and a *PAX2* LOF variant and available data (Supplementary Tables S2 and S3). The uACR as a quantitative measure of albuminuria could be calculated in 20 patients with a *PAX2* LOF variant who were all affected by (cystic) KHD, and in 65 patients carrying wildtype *PAX2* diagnosed with (cystic) KHD. When comparing these patients with unilateral or bilateral (cystic) KHD not combined with PUV, albuminuria was significantly higher in patients with versus those without *PAX2* LOF variants (*P* < 0.0001, Figure 4b), suggesting a strong proteinuric effect of *PAX2* LOF variants.

### Severe Kidney Phenotypes (i.e., Cystic KHD or KA) Were Significantly More Frequent in Patients With CAKUT and the c.76dupG Variant Than in Patients With a Nonsense or Frameshift Variant in Exon 3 to 7 in the Compiled Cohort

**Figure 5:**
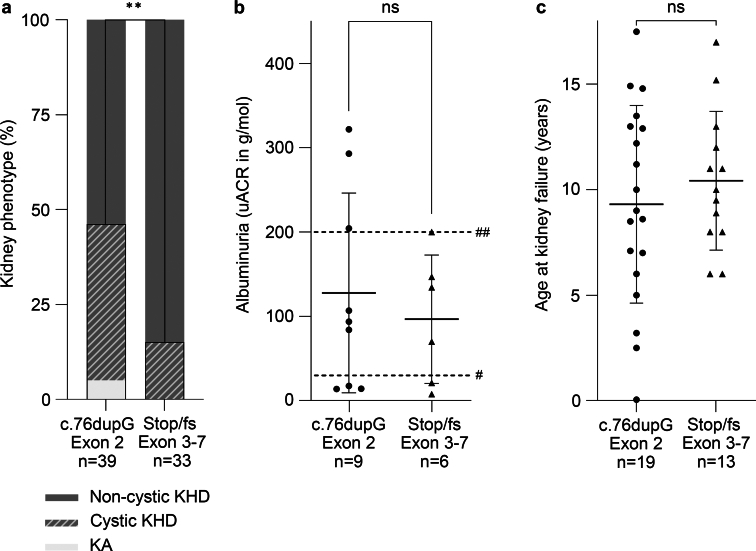
Genotype-phenotype correlations in pediatric patients with CAKUT carrying different *PAX2* LOF variants, that is, the NM_000278.5(*PAX2*):c.76dupG variant in exon 2 escaping NMD or a nonsense/frameshift (stop/fs) variant in exon 3 to 7 shown or predicted to elicit NMD. (a–c) The kidney phenotype was more severe in patients with CAKUT carrying the NM_000278.5(*PAX2*):c.76dupG variant versus an exon 3 to 7 stop/fs variant. (a) Cystic KHD or KA were significantly more frequent, (b) albuminuria levels (shown are uACRs with mean and SD) were slightly higher, and (c) kidney failure occurred at a slightly younger age (shown are mean and SD) in patients with CAKUT and a NM_000278.5(*PAX2*):c.76dupG versus exon 3 to 7 stop/fs variant. A3 albuminuria (uACR > 30 g/mol), dashed line marked with #; albuminuria in the nephrotic range (uACR > 200 g/mol), dashed line marked with ##; CAKUT, congenital anomalies of the kidney and urinary tract; cystic KHD, all cystic KHD phenotypes including multicystic dysplastic kidney and solitary kidney cysts; fs, frameshift; KA, kidney agenesis; KHD, kidney hypoplasia/dysplasia/hypodysplasia; LOF, loss-of-function; NMD, nonsense-mediated RNA decay; ns, not significant; uACR, urine albumin-to-creatinine ratio; WT, wildtype. ∗∗*P* < 0.01.

To explore genotype-phenotype correlations within the group of patients with CAKUT carrying a *PAX2* LOF variant compiled here, we compared the kidney phenotype, albuminuria levels, and age at kidney failure of 39 patients with the common c.76dupG variant located in exon 2 shown to escape NMD, and 33 patients with a nonsense or frameshift (stop/fs) variant located in exon 3 to 7 predicted or shown to elicit NMD (Figure 1b). Cystic KHD or KA were significantly more frequent in patients with CAKUT and the c.76dupG variant than with a stop/fs variant in exon 3 to 7 (18/39 [46%] vs. 5/33 [15%], *P* = 0.0058) (Figure 5). Albuminuria was on average more severe, although not significantly, in c.76dupG versus exon 3 to 7 stop/fs variant carriers (*P* = 0.5821) (Figure 5). These data suggest a more severe CAKUT phenotype in patients carrying the c.76dupG variant.

## Discussion

*PAX2*, a key kidney development gene expressed in all stages of kidney development and the mature nephron (https://www.wikipathways.org/pathways/WP5236.html), is among the genes most frequently mutated in cases with CAKUT from the fetus (4.4%)^13^ to the adult (1.8%).^14^ In this study, we focused on pediatric patients with CAKUT and their families and on *PAX2* LOF variants classified as likely pathogenic or pathogenic, and we detected heterozygous likely pathogenic or pathogenic *PAX2* LOF variants in 7 of 301 cases (2.3%). *PAX2* LOF variants were enriched (6.7%) in pediatric patients with bilateral (cystic) KHD.

Consistently, KHD without or, less frequently, with cysts was a hallmark phenotype in 97% of pediatric patients with CAKUT and a *PAX2* LOF variant from our cohort and the literature^15^^,^^27^, ^28^, ^29^^,^^31^, ^32^, ^33^, ^34^, ^35^, ^36^, ^37^, ^38^ compiled here, with a significantly higher prevalence compared with 59% in pediatric patients with CAKUT without a *PAX2* LOF variant from our cohort. These findings corroborate previous reports of patients with RCS or isolated CAKUT and *PAX2* variants.^6^^,^^7^^,^^12^

Albuminuria, a sign of glomerular damage, is part of the phenotype spectrum observed in CKD due to CAKUT. Here, we show that albuminuria was highly prevalent in patients with CAKUT carrying a *PAX2* LOF variant, affecting 81% of cases, making albuminuria the second hallmark kidney manifestation of carriers of a *PAX2* LOF variant. Similar to a report in Japanese patients with RCS,^39^ albuminuria was more severe in patients with (cystic) KHD and a *PAX2* variant versus those without a *PAX2* variant. Furthermore, *PAX2* variants have now been detected in patients with adult- and childhood-onset FSGS marked by significant proteinuria.^10^^,^^11^ Comparably, 2 adults in our study carrying the same *PAX2* LOF variant as their child with (cystic) KHD presented with severe albuminuria or FSGS, not (cystic) KHD. Both (cystic) KHD and FSGS were observed in 3 Korean patients with RCS carrying the *PAX2*:c.76dupG variant that were investigated by kidney biopsy,^15^ showing that *PAX2* variants may affect both kidney structure and glomerular integrity in some patients. Taken together, these data suggest a proteinuric effect of pathogenic *PAX2* variants in patients with (cystic) KHD more than the expected level of albuminuria due to (cystic) KHD, and even in carriers of a *PAX2* LOF variant without (cystic) KHD according to kidney ultrasound.

Proteinuria has been associated with CKD progression and the development of kidney failure in children with CAKUT^40^ and adults with CKD.^41^ In a randomized trial on children with CKD mostly due to CAKUT or glomerulopathies, lowering proteinuria by renin-angiotensin-aldosterone system inhibition has been associated with long-term preservation of kidney function.^42^ In line with these data, proteinuria decreased markedly after treatment with an angiotensin receptor antagonist in Chinese twins with FSGS and the *PAX2*:c.76dupG variant.^43^ Here, a massive decrease in albuminuria was observed in the patient with KHD carrying the *PAX2*:c.496+4A>G variant after treatment with an angiotensin-converting enzyme inhibitor starting at the age of 5 years. Due to severe albuminuria in most pediatric patients with CAKUT and a *PAX2* variant and a median age at kidney failure of 9.5 years, close monitoring starting in infancy and antiproteinuric measures should be considered and may be particularly effective. Monitoring *PAX2* variant carriers is still relevant in adulthood because the age at kidney failure in patients with RCS with a *PAX2* variant ranges from 0 to 79 years.^6^

Haploinsufficiency is the presumed pathomechanism in disorders caused by heterozygous *PAX2* aberrations.^6^ Consistently, the consequences of the c.496+4A>G and c.685C>T variants located in *PAX2* intron 4 and exon 6 were not detected in urine RNA of the respective patient, indicating NMD and suggesting reduced PAX2 protein dosage as pathomechanism. However, the c.76dupG variant located in *PAX2* exon 2 was identified on the RNA level showing that the mutant RNA is not degraded by NMD. Therefore, the c.76dupG variant should result in a severely truncated protein that could have a dominant-negative effect, for instance by retaining the ability to bind DNA or other proteins but otherwise being nonfunctional. Similarly, in mice heterozygous for this same variant (*PAX2*:c.76dupG, also known as G619 insertion), the mutant allele was identified at the RNA level together with the wildtype allele.^9^ The NMD escape of the *PAX2*:c.76dupG variant may be permitted because the generated premature termination codon is located within approximately 150 nucleotides of the translation initiation codon,^30^ and may apply to other LOF variants located in exon 1 or 2. First evidence for a possible dominant-negative effect of certain *PAX2* variants came from functional analysis of FSGS-associated *PAX2* missense variants, showing that mutant PAX2 may have an altered interaction with PAX2 repressor proteins, resulting in enhanced repressor activity with an effect beyond PAX2 haploinsufficiency.^10^

If *PAX2* variants do vary with respect to eliciting a dominant-negative effect, one may expect to find genotype-phenotype correlations even within the group of *PAX2* LOF variants. And indeed, the *PAX2*:c.76dupG variant with a possible dominant-negative effect is significantly more frequently associated with a severe kidney phenotype, that is, cystic KHD or KA, than nonsense or frameshift variants in exon 3 to 7 of *PAX2* that most likely lead to haploinsufficiency only. Moreover, albuminuria was, on average, more pronounced in patients with CAKUT carrying the c.76dupG variant versus an exon 3 to 7 nonsense or frameshift variant in *PAX2*, although not significantly so, possibly because of the small number of cases with available data. Previous genotype-phenotype correlations have suggested that FSGS-associated *PAX2* variants are more likely missense variants,^10^^,^^11^ and RCS-associated *PAX2* variants tend to be LOF variants.^6^^,^^7^^,^^15^

In our cohort, full penetrance for a kidney phenotype was observed in carriers of a *PAX2* LOF variant. In line with our data, the penetrance of *PAX2* variants with respect to abnormal kidney structure or function was previously reported to be 92%.^6^ However, variable expressivity was also observed in our study. Two pediatric patients carrying the *PAX2:*c.76dupG variant presented at the age of 4 years with either CKD stage G3b and A3 albuminuria or CKD stage G2 and A2 albuminuria. Moreover, 2 parents carrying the *PAX2*:c.56dupG or c.76dupG variants, both with a possible dominant-negative effect, were not affected by CAKUT, but presented with either A3 albuminuria or FSGS and adult-onset kidney failure. These findings are in line with previous data on the variable kidney manifestations, including CAKUT and FSGS, and kidney function in patients with the *PAX2*:c.76dupG variant.^11^^,^^15^^,^^35^^,^^44^ In our cohort, most patients with CAKUT and *PAX2* LOF variants required kidney transplantation and living donor evaluation of relatives. In this context, it should be considered that approximately 50% of the parents carry the *PAX2* LOF variant and are almost certain to develop an abnormal kidney function, that is progressive proteinuria and/or reduced estimated glomerular filtration rate, although they may have a normal kidney ultrasound at the time of living donor evaluation.

Limitations of this study include the selection bias introduced when using patients from our cohort and those previously published^15^^,^^27^, ^28^, ^29^^,^^31^, ^32^, ^33^, ^34^, ^35^, ^36^, ^37^, ^38^ because symptomatic cases, particularly those that are severely affected, are more likely to receive a diagnostic work-up, including genetic testing, leading to the diagnosis of CAKUT with or without a *PAX2* variant; whereas carriers of a *PAX2* variant with less severe phenotypes may escape detection. Furthermore, no functional experiments were done here to support our hypothesis of a possible dominant-negative effect of the *PAX2:*c.76dupG variant.

In conclusion, this study identified (cystic) KHD and severe albuminuria as hallmark kidney manifestations in 104 pediatric carriers of a *PAX2* LOF variant reported here, making close monitoring and antiproteinuric measures advisable. Particularly severe kidney manifestations, cystic KHD and KA, were detected in patients with CAKUT carrying the *PAX2:*c.76dupG variant not degraded by NMD and possibly eliciting a dominant-negative effect compared with patients with a *PAX2* nonsense or frameshift variant in exon 3 to 7 with haploinsufficiency as the pathomechanism. The high penetrance and variable expressivity with respect to kidney manifestations of *PAX2* LOF variants combined with inheritance from a parent in at least half of the patients, highlights the need for *PAX2* variant screening in living related donors of patients with CAKUT carrying a *PAX2* LOF variant.

## Disclosure

JHB received honoraria from Novartis and Alexion for lectures. DH is president of the European Society of Paediatric Nephrology, on the board of directors of the European Kidney Health Alliance, cochair of a working group of the European Rare Kidney Disease Reference Network, and a council member of the International Pediatric Nephrology Association; received grants from Kyowa Kirin, Chiesi, and the Cystinosis Research Foundation, received honoraria for lectures from Biologix, Chiesi, Kyowa Kirin, Recordati, and Sandoz; and is on a Kyowa Kirin advisory board. All the other authors declared no competing interests.

## Supplementary Material

Supplementary File (PDF and XLSX) **Supplementary Results.** Case reports of our 7 pediatric patients carrying *PAX2* loss-of-function (LOF) variants. **Figure S1.** Electropherograms of *PAX2* LOF variants in our patients with CAKUT and their segregation. **Figure S2.** Composition of the test and comparator group used in this study. **Table S1.** Oligonucleotides used for amplification and Sanger sequencing. **Table S2.** Characteristics of 104 pediatric patients with CAKUT with *PAX2* LOF variants from our cohort and 12 reviewed publications. **Table S3.** Clinical and genetic data of each pediatric patient with CAKUT with a *PAX2* LOF variant from our cohort and 12 reviewed publications (*n* = 104) (XLSX). **Table S4.** Clinical data of each pediatric patient with CAKUT with wildtype *PAX2* from our cohort (*n* = 294) (XLSX). **Supplementary References.** STROBE Checklist.
