# Supplementary material for: Presentation of Patients With Congenital Anomalies of the Kidney and Urinary Tract and PAX2 Loss-of-Function Variants and Implications for Clinical Management
Source: Kidney Int Rep. 2025 Sep 3;10(11):4041–54. doi: 10.1016/j.ekir.2025.08.037 (PMC12640069; doi:10.1016/j.ekir.2025.08.037)
Supplement: Supplementary File (PDF and XLSX) — Supplementary Results. Case reports of our 7 pediatric patients carrying PAX2 loss-of-function (LOF) variants. Figure S1. Electropherograms of PAX2 LOF variants in our patients with CAKUT and their segregation. Figure S2. Composition of the test and comparator group used in this study. Table S1. Oligonucleotides used for amplification and Sanger sequencing. Table S2. Characteristics of 104 pediatric patients with CAKUT with PAX2 LOF variants from our cohort and 12 reviewed publications. Table S3. Clinical and genetic data of each pediatric patient with CAKUT with a PAX2 LOF variant from our cohort and 12 reviewed publications (n = 104) (XLSX). Table S4. Clinical data of each pediatric patient with CAKUT with wildtype PAX2 from our cohort (n = 294) (XLSX). Supplementary References. STROBE Checklist. [file mmc1.pdf]

Supplementary Material

Presentation of Patients With Congenital Anomalies of the Kidney and Urinary Tract and *PAX2* Loss-of-Function Variants and Implications for Clinical Management

Leonie Greipel[1,2,3], Helge Martens[1], Lina Werfel[1,2], Ann Christin Gjerstad[4], Bernd Auber[1], Robert Geffers[5], Jan H. Bräsen[6], Augustina Jankauskiene[7], Anna Bjerre[4], Nele Kanzelmeyer[2], Dieter Haffner[2], Ruthild G. Weber[1]

[1]Department of Human Genetics, Hannover Medical School, Hannover, Germany;

[2]Department of Pediatric Kidney, Liver, Metabolic and Neurological Diseases, Hannover Medical School, Hannover, Germany;

[3]PRACTIS Clinician Scientist Program, Dean's Office for Academic Career Development, Hannover Medical School, Germany;

[4]Division of Paediatric and Adolescent Medicine, Oslo University Hospital, Oslo, Norway;

[5]Genome Analytics Research Group, Helmholtz Centre for Infection Research, Braunschweig, Germany;

[6]Nephropathology, Institute of Pathology, Hannover Medical School, Hannover, Germany;

[7]Pediatric Center, Institute of Clinical Medicine, Vilnius University, Vilnius, Lithuania.

Correspondence to: Ruthild G. Weber, Department of Human Genetics OE 6300, Hannover Medical School, Carl-Neuberg-Str. 1, 30625 Hannover, Germany, Phone: +49 511 5327751, Fax: +49 511 53218520, E-mail: weber.ruthild@mh-hannover.de

D. Haffner and R.G. Weber contributed equally as senior authors.

Supplementary Results

Case reports of our 7 pediatric patients with CAKUT carrying a *PAX2* loss-of-function (LOF) variant

N038-II.01: NM_000278.5(*PAX2*):c.56dupG p.(Val20fs*34), paternally inherited

The currently 19-year-old female patient was born at 38 weeks of gestation weighing 3346g. She presented with bilateral kidney hypodysplasia as well as extrarenal features, including severe myopia, macrocephaly, high-arched palate, and short narrow palpebral fissures. She had early-onset kidney failure and received a kidney transplant at the age of 4 years and 3 months. A second kidney transplantation was performed at the age of 9 years due to chronic rejection. In her father, N038-I.01, who also carried the *PAX2* LOF variant, kidney ultrasound was unremarkable, but focal segmental glomerulosclerosis was diagnosed after kidney biopsy. He developed kidney failure in early adulthood and received kidney transplantation. He also presented with visual impairment.

A011-II.01: NM_000278.5(*PAX2*):c.76delG p.(Val26fs*3), *de novo*

The currently 25-year-old male patient was born with bilateral kidney hypodysplasia, a single kidney cyst in the right kidney, and bilateral pes calcaneus at 33 weeks of gestation weighing 1200g. Due to severely impaired kidney function, including A3 albuminuria, he was listed for kidney transplantation at the age of 2 years, and received a kidney transplant at the age of 4 years and 9 months. After transplantation, he was affected by post-transplant lymphoproliferative disorder and recurrent pneumonia. Additionally, his eyesight is impaired by hyperopia of at least 8 diopters.

A042-II.03: NM_000278.5(*PAX2*):c.76dupG p.(Val26fs*28), *de novo*

The currently 20-year-old female patient was diagnosed with bilateral kidney hypodysplasia and hydronephrosis of the right kidney in early childhood. Her medical records were available from the age of 13 years, when she presented with chronic kidney disease (CKD) of stage G3b and A3 albuminuria. She was listed for transplantation at the age of 14 years and 11 months,

and received a kidney transplant one year later. After transplantation, she developed glucose intolerance. Additionally, she presented with severe myopia, luxation of the hip, and abdominal hernia requiring 3 operations. She also received otoplasty at the age of 5 years.

B005-II.02: NM_000278.5(*PAX2*):c.76dupG p.(Val26fs*28), *de novo*

The currently 15-year-old male patient was born at 37+0 weeks of gestation weighing 2990g. He was diagnosed with bilateral kidney hypodysplasia with multiple cysts of the right kidney and bilateral vesicoureteral reflux. His kidney function declined in early childhood from CKD stage G3a to G4. At the age of 8 years and 6 months, he was listed for kidney transplantation, and received a transplant one year later. Additionally, he presented with bilateral optic nerve dysplasia, amaurosis of the right eye, and cryptorchidism, and received fundoplication.

B061-II.01: NM_000278.5(*PAX2*):c.76dupG p.(Val26fs*28), maternally inherited

The currently 4-year-old male patient was born at 39+5 weeks of gestation weighing 2720g. Prenatally, left-sided kidney hypodysplasia and right-sided multicystic dysplastic kidney were diagnosed by ultrasound. At the age of 5 months, bilateral vesicoureteral reflux was detected. There were no extrarenal features. His total native kidney volume is presently normal and his kidney function is mildly impaired with CKD stage G2 and A2 albuminuria. His mother, B061-I.02, was identified to carry the same *PAX2* LOF variant. By ultrasound, her kidneys were unremarkable. A further kidney work-up showed A3 albuminuria (urine albumin-to-creatinine ratio of 44 g/mol). She also presented with myopia, as did her mother, the maternal grandmother of B061-II.01.

N075-III.03: NM_000278.5(*PAX2*):c.496+4A>G, paternally inherited

The currently 8-year-old female patient was born at 36+4 weeks of gestation weighing 3390g. By ultrasound, she was diagnosed with bilateral kidney hypodysplasia. Kidney biopsy revealed oligomeganephronia. Additionally, she presented with severe albuminuria in the nephrotic range, which decreased after treatment with ACE inhibitors. Her kidney function had declined

to CKD stage G3a at the age of 8 years. There were no extrarenal features. Her father, N075-II.02, who also carried the *PAX2* LOF variant, developed kidney failure at 16 years of age due to an undefined kidney disorder and received kidney transplantation. Furthermore, her paternal uncle and grandfather developed kidney failure because of undefined kidney disorders. Both were not available for genetic testing.

C018-II.01: NM_000278.5(*PAX2*):c.685C>T p.(Arg229*), not maternally, probably paternally inherited

The currently 16-year-old female patient was diagnosed with bilateral kidney dysplasia at the age of 6 years. Her kidney function is presently mildly impaired with CKD stage G2 and A2 albuminuria. Her father, C018-I.01, was diagnosed with bilateral kidney hypodysplasia, developed kidney failure, received dialysis at the age of 36 years, and died at 40 years of age. Therefore, genetic testing was not possible. Both the patient and her father presented with myopia. Because of the kidney and ocular phenotype of the father, he is presumed to have carried the *PAX2* LOF variant and passed it on to his daughter. Kidney ultrasound in her mother, who does not carry the *PAX2* LOF variant, was unremarkable.

Supplementary Figures

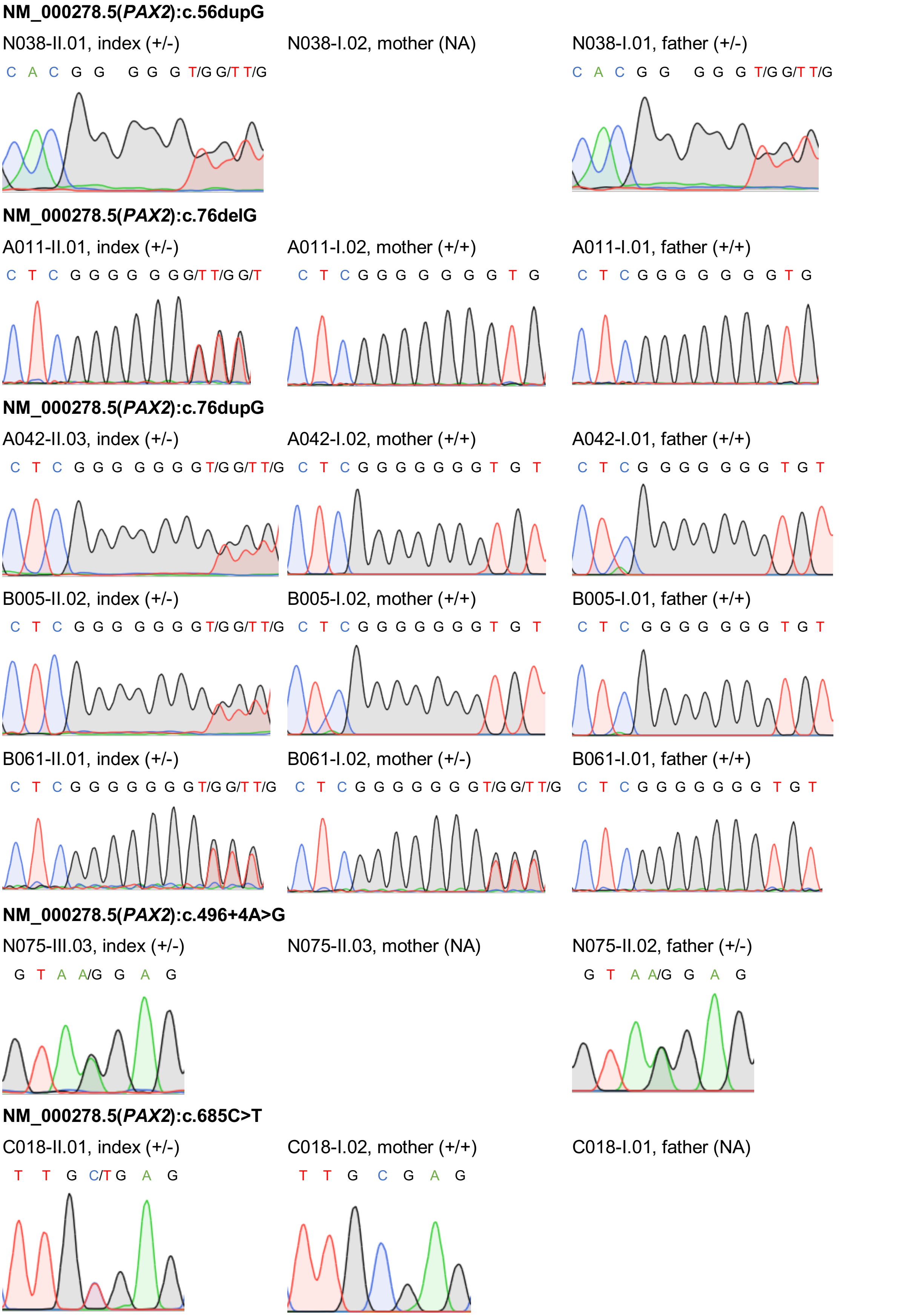

Supplementary Figure S1. Electropherograms of *PAX2* LOF variants in our patients with CAKUT and their segregation. Next-generation sequencing data were verified by targeted Sanger sequencing. NA, not available

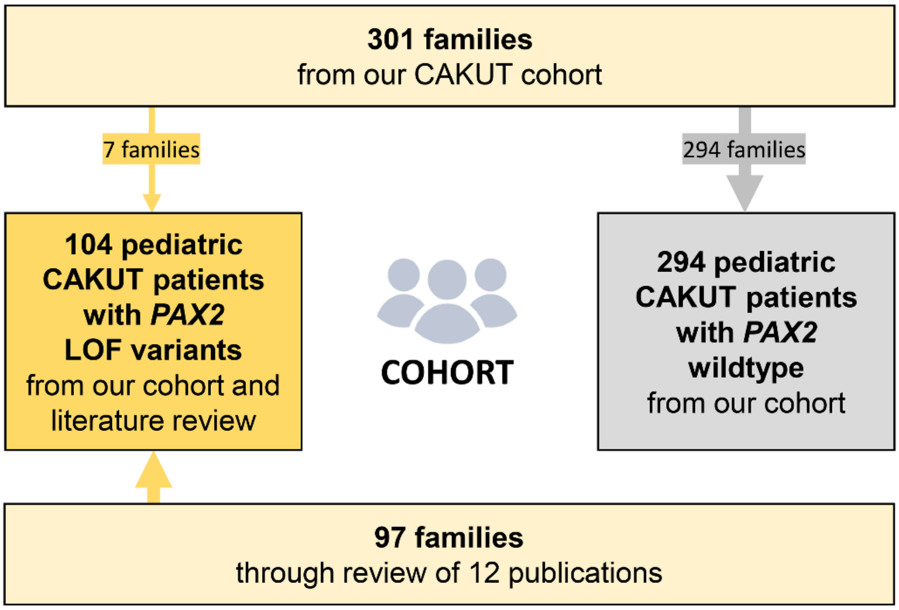

Supplementary Figure S2. Composition of the test and comparator group used in this study. The test group of 104 pediatric CAKUT patients with *PAX2* LOF variants was compiled from our CAKUT cohort (7 cases) and 12 publications (97 cases) [S2-S13]. The comparator group consisted of the remaining 294 CAKUT patients with wildtype *PAX2* from our cohort. LOF, loss-of-function

Supplementary Tables

Supplementary Table S1. Oligonucleotides used for amplification and Sanger sequencing

Designation	Sequence (5' to 3')
**Amplification and Sanger sequencing of genomic DNA**	
*PAX2*_Exon2_forward_1	CGC GGA CCC TGA CTA ATG
*PAX2*_Exon2_reverse_1	CTG GCC GGG GAA GTA GG
*PAX2*_Exon4_forward	GAG CAG ATG GAT GAG GAA ATC
*PAX2*_Exon4_reverse	GTT GGG CTC TGG ACC TCA AG
*PAX2*_Exon6_forward	CCT CAG CCA GAT CTC TGA GG
*PAX2*_Exon6_reverse	CAC AGA ACA CAC GTG TGT AAC
**Amplification and Sanger sequencing of cDNA**	
*PAX2*_UTR_forward	CTC CTC AAG TCC TGA AGT TGA G
*PAX2*_Exon2_reverse*	GGG CCA GCT CCA CGA TG
*PAX2*_Exon3_forward	CAA AGT GGT GGA CAA GAT TGC
*PAX2*_Exon5_reverse	GTT TCC TCT TCT CAC CAT TGG AG
*PAX2*_Exon6_reverse	GAT CCA AAG CTT CCA GCT GC

*Oligonucleotide sequence has been previously reported in Yamamura et al. [S1]

Supplementary Table S2. Characteristics of 104 pediatric patients with CAKUT and a *PAX2* LOF variant of our cohort and 12 reviewed publications

Studies	Total	Negrisolo et al. [S2]	Iwafuchi et al. [S3]	Deng et al. [S4]	Rossanti et al. [S5]	Yang et al. [S6]	Chang et al. [S7]	Domingo -Gallego et al. [S8]	Xiong et al. [S9]	Amma-yappan et al. [S10]	Ben-Moshe et al. [S11]	Liu et al. [S12]	Kim et al. [S13]	Our study
**Number of patients**	104	2	1	6	10	18	3	4	7	1	2	23	20	7
**Male / female**	64/104 (62%)/ 40/104 (38%)	1 / 1	1 / 0	3 / 3	6 / 4	10 / 8	2 / 1	2 / 2	6 / 1	1 / 0	2 / 0	12 / 11	14 / 6	4 / 3
**CKD stage G5# or kidney failure**	56/104 (54%)	2	1	1	3	12	2	1	3	1	2	14	10	4
**Median age (years) at CKD stage G5#**	9.5	NA	7.0	11.2	NA	10.0	4.5	8.0	13.2	17.0	8.0	NA	9.2	6.4
***PAX2* LOF variant *de novo* / inherited**	54/67 (81%) / 13/67 (19%)	1 / 0	0 / 1	4 / 0	2 / 3	16 / 1	0 / 1	3 / 1	6 / 1	1 / 0	NA	18 / 2	NA	3 / 3
**Bilateral KHD**	56/104 (54%)	1	1	3	6	15	0	3	6	1	0	16	0	4
**Bilateral cystic KHD**	11/104 (11%)	0	0	1	1	2	3	0	1	0	0	0	0	3
**Uni- or bilateral KHD**	15/104 (14%)	1	0	0	0	0	0	0	0	0	2	2	10	0
**Uni- or bilateral cystic KHD**	13/104 (13%)	0	0	0	1	1	0	1	0	0	0	0	10	0
**Unilateral KHD**	4/104 (4%)	0	0	0	1	0	0	0	0	0	0	3	0	0
**Unilateral cystic KHD**	2/104 (2%)	0	0	2	0	0	0	0	0	0	0	0	0	0
**Other kidney phenotype**	3/104 (3%)	0	0	0	1	0	0	0	0	0	0	2	0	0
**Albuminuria or pro-teinuria (yes / no)**	38/47 (81%) / 9/47 (19%)	NA	1 / 0	6 / 0	NA	NA	2 / 0	1 / 0	7 / 0	1 / 0	NA	3 / 0	11 / 9	6 / 0
**Ocular phenotype (yes / no)**	64/96 (67%) / 32/96 (33%)	2 / 0	NA	3 / 1	6 / 3	10 / 4	3 / 0	2 / 2	2 / 5	1 / 0	0 / 2	12 / 11	18 / 2	5 / 2
**Other (yes / no)**	39/82 (48%) / 43/82 (52%)	1 / 0	NA	2 / 4	6 / 4	8 / 10	3 / 0	1 / 3	4 / 3	1 / 0	2 / 0	6 / 17	NA	5 / 2

#According to the KDIGO 2024 guidelines [S14].
Cystic KHD, all cystic KHD phenotypes including multicystic dysplastic kidney and solitary kidney cysts not combined with posterior urethral valves; KHD, kidney hypoplasia/dysplasia/hypodysplasia not combined with posterior urethral valves; LOF, loss-of-function; NA, not available

Supplementary Table S3. Clinical and genetic data of each pediatric patient with CAKUT and a *PAX2* LOF variant of our cohort and 12 reviewed publications (n=104) (see separate Excel file)

Supplementary Table S4. Clinical data of each pediatric patient with CAKUT and wildtype *PAX2* of our cohort (n=294) (see separate Excel file)

Supplementary References

S1. Yamamura Y, Furuichi K, Murakawa Y, et al. Identification of candidate PAX2-regulated genes implicated in human kidney development. *Sci Rep* 2021;11:9123. DOI: 10.1038/s41598-021-88743-1

S2. Negrisolo S, Benetti E, Centi S, et al. PAX2 gene mutations in pediatric and young adult transplant recipients: kidney and urinary tract malformations without ocular anomalies. *Clin Genet* 2011;80:581-585. DOI: 10.1111/j.1399-0004.2010.01588.x

S3. Iwafuchi Y, Morioka T, Morita T, et al. Diverse Renal Phenotypes Observed in a Single Family with a Genetic Mutation in Paired Box Protein 2. *Case Rep Nephrol Dial* 2016;6:61-69. DOI: 10.1159/000445679

S4. Deng H, Zhang Y, Xiao H, et al. Diverse phenotypes in children with PAX2-related disorder. *Mol Genet Genomic Med* 2019;7:e701. DOI: 10.1002/mgg3.701

S5. Rossanti R, Morisada N, Nozu K, et al. Clinical and genetic variability of PAX2-related disorder in the Japanese population. *J Hum Genet* 2020;65:541-549. DOI: 10.1038/s10038-020-0741-y

S6. Yang X, Li Y, Fang Y, et al. Phenotypic spectrum and genetics of PAX2-related disorder in the Chinese cohort. *BMC Med Genomics* 2021;14:250. DOI: 10.1186/s12920-021-01102-x

S7. Chang YM, Chen CC, Lee NC, et al. PAX2 Mutation-Related Renal Hypodysplasia: Review of the Literature and Three Case Reports. *Front Pediatr* 2022;9:765929. DOI: 10.3389/fped.2021.765929

S8. Domingo-Gallego A, Pybus M, Bullich G, et al. Clinical utility of genetic testing in early-onset kidney disease: seven genes are the main players. *Nephrol Dial Transplant* 2022;37:687-696. DOI: 10.1093/ndt/gfab019

S9. Xiong HY, Shi YQ, Zhong C, et al. Detection of De Novo PAX2 Variants and Phenotypes in Chinese Population: A Single-Center Study. *Front Genet* 2022;13:799562. DOI: 10.3389/fgene.2022.799562

S10. Ammayappan SK, Rajagopalan A, Arunachalam J, et al. A case of renal coloboma syndrome. *J Nephrol* 2023;36:233-235. DOI: 10.1007/s40620-022-01383-0

S11. Ben-Moshe Y, Shlomovitz O, Atias-Varon D, et al. Diagnostic Utility of Exome Sequencing Among Israeli Children With Kidney Failure. *Kidney Int Rep* 2023;8:2126-2135. DOI: 10.1016/j.ekir.2023.07.019

S12. Liu JL, Wang XW, Liu CH, et al. Genetic spectrum of CAKUT and risk factors for kidney failure: a pediatric multicenter cohort study. *Nephrol Dial Transplant* 2023;38:1981-1991. DOI: 10.1093/ndt/gfac338

S13. Kim, JH, Ahn, YH, Jang, Y, et al. Genotype of PAX2-related disorders correlates with kidney and ocular manifestations. *Eur J Hum Genet.* 2025;33:441-450. DOI: 10.1038/s41431-025-01822-z

S14. Kidney Disease: Improving Global Outcomes (KDIGO) CKD Work Group. KDIGO 2024 Clinical Practice Guideline for the Evaluation and Management of Chronic Kidney Disease. *Kidney Int.* 2024;105:117-314. DOI: 10.1016/j.kint.2023.10.018
